# Mr. Hill, on Cancer

**Published:** 1804-11-01

**Authors:** Geo. N. Hill

**Affiliations:** Chester


### *To the Editors of the Medical and Physical Journal.*

GENTLEMEN,

THE well-known axiom, that "prevention of disease is better than cure," cannot be more forcibly illustrated, than in the melancholy, horrible affection, which is the object of concern in the present paper.

The great importance of the subject of glandular obstruction and consequent disease, will ensure me the attention of every chirurgical artist, particularly at this period, when the laudable spirit of scrutinizing enquiry and ardent research so perseveringly exerts its never-ceasing efforts for the public good. The very name of cancer carries along with it the ultimatum of misery; nor does the dread it always inspires, fail to contribute largely and rapidly to its production; accelerates its increase and mortal termination. To lessen this dread, to calm the agony of anticipated suffering, and to afford a reasonable hope that such evils may frequently be altogether prevented, are objects of interesting magnitude.

The conduct of young men attending public hospitals, and surgical lectures, confirms the opinion that, unhappily, it has too often been deemed more honourable to the surgeon to perform some of the greater operations of his art with a certain dexterity and dispatch, and thereby remove some loathsome and extensive disease, than to possess abilities capable of superseding the necessity of so frequently operating at all, and by the aid of medical surgery preventing altogether the progressive advances of diseases to that point which renders an operation the *unicum remedium;* but in my humble opinion, there can be no just comparison made between the two practitioners who take these opposite routs, to acquire laudable fame and honorable repute. The man who is capable, from his acquaintance with the resources of Nature, from his sagacity and patient attention, to save the carious leg of a fellow-creature from amputation, and cure it, most certainly ought to rank higher in the scale of merit, than he whose acme of ability consists in a quick and dextrous mode of depriving the sufferer of his diseased limb.

Much has been written, and much said on this great subject, by able persons; still it is certain, that operations are yet oftener boldly and needlessly performed, than timidly and injuriously avoided. From which of these sources sources mankind have endured most mischief, I shall not attempt to decide; it is sufficient for my present purpose to observe, that it appears to have become quite fashionable, immediately to remove every indurated gland from every part of the body, almost wherever situated, great or small, easy or painful, of long or short duration, in every period of life, in all constitutions; in short, under all or whatever circumstances. No sooner does an unfortunate female (particularly) perceive that she has a lump, as it is called, in her breast, or elsewhere, which was never before recognised, than instantaneously the trumpet of alarm is sounded; terror follows close, and too often brings in its train that positive evil, which, but for sudden and often unfounded suspicion, might have never happened, or at least with ease have been avoided. I am not clearly convinced but that sometimes the conduct of surgeons (especially those who, on all occasions, manifest a strong predilection for the knife) has greatly contributed to produce this mischievous state of matters, by hastily pronouncing almost every enlarged or hardened gland as likely one day or other to produce cancer; and that such gland cannot too speedily be removed.

Now, in order fully to justify this prognosis, and such a mode of doing business, it appears to me necessary that such practitioners should, in the first place, always be able to say exactly what swelling of this description is scirrhous, and may ultimately become cancerous. Secondly, for the satisfaction of his own feelings, and the welfare of his patient, to afford indubitable evidences that his opinion is founded in truth. Unless these rules can always be fulfilled, it has long seemed to me unjustifiable practice to be so hasty in removing parts which might either never be mischievous, or be restored to healthy action by gentle means; many instances have occurred to me, and doubtless a greater number to many of my brethren, where surgeons of considerable ability have pronounced, that nothing but cutting out a tumour could prevent a cancer, when such tumour has afterwards disappeared either from no medical or surgical treatment whatever, from the incantations of some mumbling old Sybil, or the nostrums of some pompous empiric. Whenever this happens, the result does not yield a very grateful feeling to the professional man. Some years ago I removed several enlarged glands from different parts of the body, for persons whose minds had become so terribly irritated, that nothing short of instant extirpation could appease their fears, I am now well assured that most of of these ought never to have been removed at all, that is to say, by the scalpel. Of late years many cases have I seen of a similar complexion, that have undergone condemnation, relieved and cured by a steady perseverance in the use of appropriate means, when the mind could be brought to rely on such means with that confidence which so powerfully tends to promote the salutary object in view. But after what has been advanced on this side the question, let it not be imagined for an instant, that I mean to assert any thing which shall in the remotest degree tend to induce a false security in the minds of those who are so unfortunate as to labour under a scirrhosity in any part of the body, or prevent the timely use of the knife when it is absolutely necessary; still I contend, that the longer persons thus affected are kept ignorant of the true nature of their disease, (and it is equally the case with regard to many other affections) the greater is the chance of affording them solid assistance by suitable means.

Can it, I would ask, be supposed for a moment, that one of the tumours which are said to be removed without cutting, by the numerous pretenders which infest our country, was really a scirrhous gland or congeries of glands, and which would one day form legitimate cancer? On the contrary, we may truly affirm, that their subsidence was an incontrovertible proof of their not being scirrhous, and of course that they never would have been cancerous; it follows then, that more attention ought to be given to decide the great question, what enlarged gland is reducible to health again, and what is not. I do not hesitate to aver, that it is not every one which is very hard, considerably enlarged, somewhat knotty, or unequally surfaced, and even of some considerable standing, which ought, nolens volens, to be cut out, unless other circumstances correspond to determine, and clearly justify such a modus curandi. But we need not approach so near the precipice of danger; almost every man conversant in practice, is able to satisfy himself if he chooses, that numbers of hard, indolent, easy, unattached, glandular tumours, hastily designed for removal by the knife or caustic, may by other means be gently and safely dispersed, and diseased action finally removed. In short, means may be used to cause the system effectually to do that in an early, quiet, easy stage of the obstruction, which it in vain attempts to bring about when the tumour is become farther advanced, uneasy, or, perhaps, in the first stage of inflammation.

I am

I am not unaware of the difference of opinion existing between deservedly great men on this subject, and perhaps the scale may turn against me in the present state of practice; but, on the other hand, it would savour of the worst species of slavery for any man, honoured by a share of public confidence in the medical profession, to hesitate a moment in avowing a difference of opinion from those sanctioned by the greatest of names, where truth will bear him out.

We have no certain, indisputable, decided mode of judging what is always absolute scirrhus, irresolvable by any known means; but we take it for granted that all enlarged, hard glands that are resolved were not primarily scirrhous, or ever would be ultimately cancerous; perhaps this conclusion has been somewhat too hastily formed; the powers of medicine over the worst diseases, in their infancy, are very great, which in a more advanced stage would be little or even none at all. A catalogue of chronic distempers might readily be found to prove this, where the first links in the now short chain might by judicious treatment have been straitened, and health re-established, which, where their number, strength and tortuosity is increased, are only hastened onwards to swell the catalogue of opprobriæ medicinæ.

It has been urged with much apparent force of reasoning, that it is better twenty, forty, or indeed any number of hard tumefied glands should be removed, than one remain, which would sometime become open cancer. This argument demands examination. Is the person who has undergone an operation of this kind wholly freed thereby from all future apprehensions? Do they not often argue, and that not irrationally, that what has once happened in this particular respect may happen again? Is the perpetual dread of apprehension much less prevalent in the mind, that some other glandular part may become affected, and require an operation, than that the first swelling may ultimately find no other means of relief than the knife?

The constant anxiety in the one situation cannot be less mischievous than in the other, setting aside the high probability, that out of these twenty, forty, or any indefinite number of cases, two-thirds may not be of the nature of those which, if left alone by the scalpel, will never fail to become finally cancerous; ought not every person afflicted with glandular obstructions, of whatever nature, to be at their first discovery encouraged to hope, they may be dispersed persed by timely and steady recourse to powerful, general, and local remedies? Should success follow, will any man seriously tell me that the mental feelings of the patient will not be in a far happier (of consequence healthier) state than after endurance of a painful and dreaded operation?

It will certainly be a more satisfactory issue of the business, that the affection was removed by the action of general and local remedies on the system, than by the partial action of the knife, existing afterwards in constant fear of some other part requiring a like treatment; infinitely superior to this is the consideration, that if removed by the proposed means, we may safely assure the afflicted they have not had a scirrhus at all. Contrast this truth with the effect of instantaneous, unhesitating removal of every glandular enlargement that comes under the cognizance of modern practice, under the idea that, if left unremoved, it may some day conduct the owner to an untimely sepulchre. Or, suppose this sentence, which I fear is too common, to be passed on some helpless victim of terror by a practitioner of celebrity; and fear operating too powerfully to permit his advice to be taken, such a person applies to some empirical professed cancer curer, at a distance from home; a combination of causes produce the effect of resolution on the swelling; they return without it. Is not the credit of the art concerned in this result, which has often happened, and indeed does happen almost daily, from rash unjustifiable haste in ascertaining the just nature of the ailment, the constitution, habits, &c. of the patient? How such an event accords with the feelings of the medical artist, it is needless to discuss.

How often does latent venereal taint produce glandular obstructions amongst its multifarious Protæan shapes? Do not temperate surgeons pause in their examinations of doubtful cases, before they recommend the extirpation? Generally I hope they do. That they do not always, I am well convinced from having seen foul obstinate ulcers succeed the removal of tumors which have been cured by the appropriate remedy, after resisting all the other means very considerable ability could suggest. Could this have happened had medicine been preferred to the knife? Has hasty judgment never removed a chronic abscess for a scirrhous tumour? Or, has lurking scrophula never led to error and disappointment?

In short, it is not saying too much to aver our scanty means of acquiring the true definite criteria of legitimate scirrhus; scirrhus; how necessary then is it to be always guarded in deciding on so important a matter, seeing the most sagacious have been deceived. To discriminate, it is right to pause, and consider the business in every possible view, and all its bearings, before we decide.

I am aware that dwelling on these observations is only reiterating truths, which are daily enforced in our medical schools; yet, as they have seemed of late to be thrown somewhat into the back ground, it is clear no apology is necessary. Some who may perchance read these sentiments, prepossessed with opinions diametrically opposite, boldly adhering to the maxim, that it is better practice to remove twenty doubtful swellings than one malignant one should escape; to all such let it be observed, that it is incumbent on them to save all who consult them as much anxiety of mind and pain of body as they can, and that whilst they steer clear of Scylla, to beware they do not fall into Charybdis. The cutting out an inoffensive swelled gland (comparatively speaking) must always be an operation of considerable importance to the sufferer, whatever may be its situation, or the degree of pain inflicted, inasmuch as it involves in it great previous anxiety, and, most certain, future doubts of its proving a complete preventive of the return of similar affection. I presume no man will take upon him confidently to assure a patient that the taking from him one enlarged indurated gland, will secure him from ever having another; whereas, if this induration and swelling can be removed by general and local powers, the mind becomes at once disembarrassed of every idea of cancer, near or remote; the disease was not scirrhus, it never could be cancer. It will doubtless be observed, that this proposal embraces a serious loss of time to the patient, and a trifling with his health and feelings, if at last recourse must be had to the knife. I acknowledge this would be an invincible argument against internal medicines, local remedies, and delay, were it not more specious than true; it is well known from every day's practice, that tumours may remain (in the breast for example) through the major part of a long life, without pain or disturbance; not that I wish to be understood as an advocate for the practice of letting such tumours alone, because I am well convinced, that long residence in the state before mentioned, is one principal cause of their ultimate mischief and danger; the sooner it is known what is their real nature the better. To acquire this knowledge, it is by no means necessary they should be removed, because the operations of of nature are commonly regular and consistent, and therefore no quiescent tumour ever does so suddenly become cancerously painful, and assume the decided unequivocal form of occult cancer without sufficient warning; that there is a principle of repugnant hesitation in the human system against admitting and propagating morbid poison or diseased action, is very manifest, and has been treated of by men of eminence; it follows, therefore, from very fair premises, that upon any tumour manifesting indubitable proofs of its irresoluble texture, or creating that kind of uneasiness which is well known as the forerunner of greater evil, it is, to use an homely phrase, all in good time to remove it. But it may well be asked, does it not happen that this point of time proves, on some occasions, too late, cancerous diathesis has taken possession of the system, and, although you have removed the focus of mischief, its radii have made sure of the destruction of the miserable wretch who has trusted his life in your unskilful hands? Such an event is most certainly and securely prevented by not waiting for its commencement; for if inflammation, the necessary precursor of absorption, has not commenced, (and without pain of a peculiar well marked and well understood kind, it cannot commence) there can be no doubt but the operation will succeed as well now as it ever would.

I repeat, then, here is a tumour of some considerable standing in the breast, originating with or without assignable cause; I endeavour to explore the causes, and having a tolerable good constitution to deal with, I commence a certain mode of removing this tumour, which, in all apparently similar cases, has been successful; but here, after a reasonable length of time has elapsed, no advances towards resolution are perceived: I am now justified in removing it by the knife; having done my duty in attempting to cure that without a painful operation, which can now be as safely and securely removed by its performance as though no such attempt had been made. Should success follow in only an equal number of cases, I conceive it will be readily allowed that benefit has been derived from delay, and the adoption of general and local remedies. I trust it will be remembered through the whole of this attempted reasoning, that the cure of cancer, without cutting, is not the great design of the business; but that it is merely an endeavour to prevent the rash removal by the knife of a great number of tumours from various parts of the body, under the idea that they will assuredly sometime ime become cancerous, and which are by many surgeons no sooner seen than condemned, sufficient pains not being always taken to ascertain their true nature; the person afflicted is immediately alarmed, and if they do not speedily yield consent to an operation, which may be unnecessary, their misery is certain, and in exact proportion to the opinion entertained of the abilities of their surgeon. On the other hand, if they do consent, the operation itself, the rules enjoined for future regimen, and mode of life, and the dread of sooner or later having to endure the same scene, form a combination of circumstances sufficiently calculated to embitter future existence, and all this in a majority of cases on no just foundation. Is such a situation to be compared with that of a sufferer under a hard glandular tumour, having it gradually removed, and healthy action restored therein? Such a result is not confined in its beneficial effects to success in the first instance, but extends to a well grounded confidence that the same means will always produce like effects in any similar situation. Are surgeons always justified in removing every swelled gland? Is it perfectly clear that such gland can be spared, without injury to the constitution, without laying the foundation of more mischief than its removal is intended to prevent; and although no immediate evil consequences may seem to arise to the system from the loss of so important a part, yet, will any one assure himself that the economy of nature is not thus primarily much deranged, and may ultimately experience great inconvenience from such a deprivation? On the whole, I infer then, that it is more beneficial to sufferers in this way, that all glandular swellings, where application for advice is adopted sufficiently early, be considered as having an highly probable chance of removal without manual operation, the very hope of such an event being calculated to produce its ratification; but if this desirable result do not follow, another, little less advantageous, will, viz. it will then be known to a certainty, whether the disease be scirrhus or not. It may be syphilitic, scrophulous, chronic abscess, or simple obstruction from some now-forgotten bruise or pressure. This knowledge once acquired, is every thing; could it be earlier acquired, it would be still more valuable: but this being so often impracticable, humanity enjoins the institution of the experiment; nor can it with truth and justice be said, that the adoption of this plan will prove a serious waste of time, a tedious procrastination of what must be done at last, a corroding suspence; for, should it fail in one point, its success is sure and determinate in another, both surgeon and patient will be no longer strangers to the nature of the affection the one has to combat, and the other endure; nor will injury to the system be sustained, not one valuable moment having been lost. As to all the good that the knife can be expected to produce, we have as much now in our power as ever we had, with a great increase of satisfaction in the use of the instrument, arising from consciousness of having done all that human ability at present affords, to prevent this dernier resort, which is certainly the greatest glory, on all occasions, a good surgeon can acquire.

To conclude, it ought to be the first wish of every practitioner, that the disease be always duly and clearly characterized, before a word be mentioned of an operation. Let it be a standing established rule, that every surgeon be enabled to say, this tumour is scirrhous, and may become cancerous; it must be removed. Were this the law of conduct, the standing practical rule, I am well convinced that numbers which now are, and in future may be, condemned to the scalpel, will never be attempted to be removed thereby at all. On the other hand, all which ought to be operated upon, would, in good time, undergo the now necessary cure. I am, &c.

*Chester, Sept.* 24, 1804. GEO. N. HILL.

P. S. I think the foundation of cancerous mammæ has often been laid at the period of weaning infants. The obstruction consequent on this situation is not removed with that scrupulous attention which it ought; the rule should be, that of never remitting the application of suitable means of resolution, until the breast resumes the situation it was in previous to its distention with milk. Some mothers always dry up one breast, either from a sunk or a flat nipple, or one prone to excoriate. This conduct has led to the production of cancer on the ceasing to bear children, or at the departure of the catamenia; to which I am certain of being right when I add, this horrible disease has often resulted from females refusing to perform the most endearing and natural office peculiar to their sex. How much then is it the duty of the humane and attentive practitioner, never to omit an opportunity of using every effort to *prevent* what he is so often doomed to regret he cannot *cure!*

